# Igf2/H19 Imprinting Control Region (ICR): An Insulator or a Position-Dependent Silencer?

**DOI:** 10.1100/tsw.2001.50

**Published:** 2001-05-18

**Authors:** Subhasis Banerjee, Alan Smallwood, Scott Lamond, Stuart Campbell, Geeta Nargund

**Affiliations:** Centre for Reproductive Medicine, Department of Obstetrics & Gynaecology, St. George's Hospital Medical School, Cranmer Terrace, University of London, London SW17 ORE, UK

**Keywords:** genomic imprinting, lgf2 and H19, insulator, CTCF, transcriptional regulation

## Abstract

The imprinting control region (ICR) located far upstream of the H19 gene, in conjunction with enhancers, modulates the transcription of Igf2 and H19 genes in an allele-specific manner. On paternal inheritance, the methylated ICR silences the H19 gene and indirectly facilitates transcription from the distant Igf2 promoter, whereas on the maternal chromosome the unmethylated ICR, together with enhancers, activates transcription of the H19 gene and thereby contributes to the repression of Igf2. This repression of maternal Igf2 has recently been postulated to be due to a chromatin boundary or insulator function of the unmethylated ICR. Central to the insulator model is the site-specific binding of a ubiquitous nuclear factor CTCF which exhibits remarkable flexibility in functioning as transcriptional activator or silencer. We suggest that the ICR positioned close to the enhancers in an episomal context might function as a transcriptional silencer by virtue of interaction of CTCF with its modifiers such as SIN3A and histone deacetylases. Furthermore, a localised folded chromatin structure resulting from juxtaposition of two disparate regulatory sequences (enhancer ICR) could be the mechanistic basis of ICR-mediated position-dependent (ICR-promoter) transcriptional repression in transgenic Drosophila.

Insulin-like growth factor 2 (*Igf2*) and *H19* genes in mouse and human are reciprocally imprinted. In the maternal chromosome, the *H19* gene is preferentially transcribed while *Igf2* is inactive, whereas in the paternal chromosome the *H19* gene is silent and *Igf2* is activated. Several years ago, we proposed [1] that transcriptional regulation of *Igf2/H19* genes could be explained by considering that *Igf2* and *H19* genes are partitioned into two topologically independent chromatin loops. In this model, the enhancer-dependent trafficking of RNA polymerase complexes to either of the two promoters was primarily controlled by DNA methylation-dependent differential condensation of the *H19* domain. In the last few years, the imprinting control region (ICR) located far upstream of the mouse/human *H19* gene has been a major focus of research because of its pivotal role in *Igf2/H19* imprinting [2,3,4,5,6,7,8] (see Fig. 1a). Earlier experiments [4] suggested that a region containing a part of ICR (–1.8 to –3.0 kb) was a transcriptional silencer (Fig. 1a). Maternal transmission of an ICR deletion revealed that these sequences could act as an activator of the *H19* gene as well as a repressor of the distant *Igf2* [5]. In a recent series of reports, ICR has been identified as an insulator [9,10,11,12]. Here we reconcile the published data and suggest that the insulator function of ICR is a position effect in

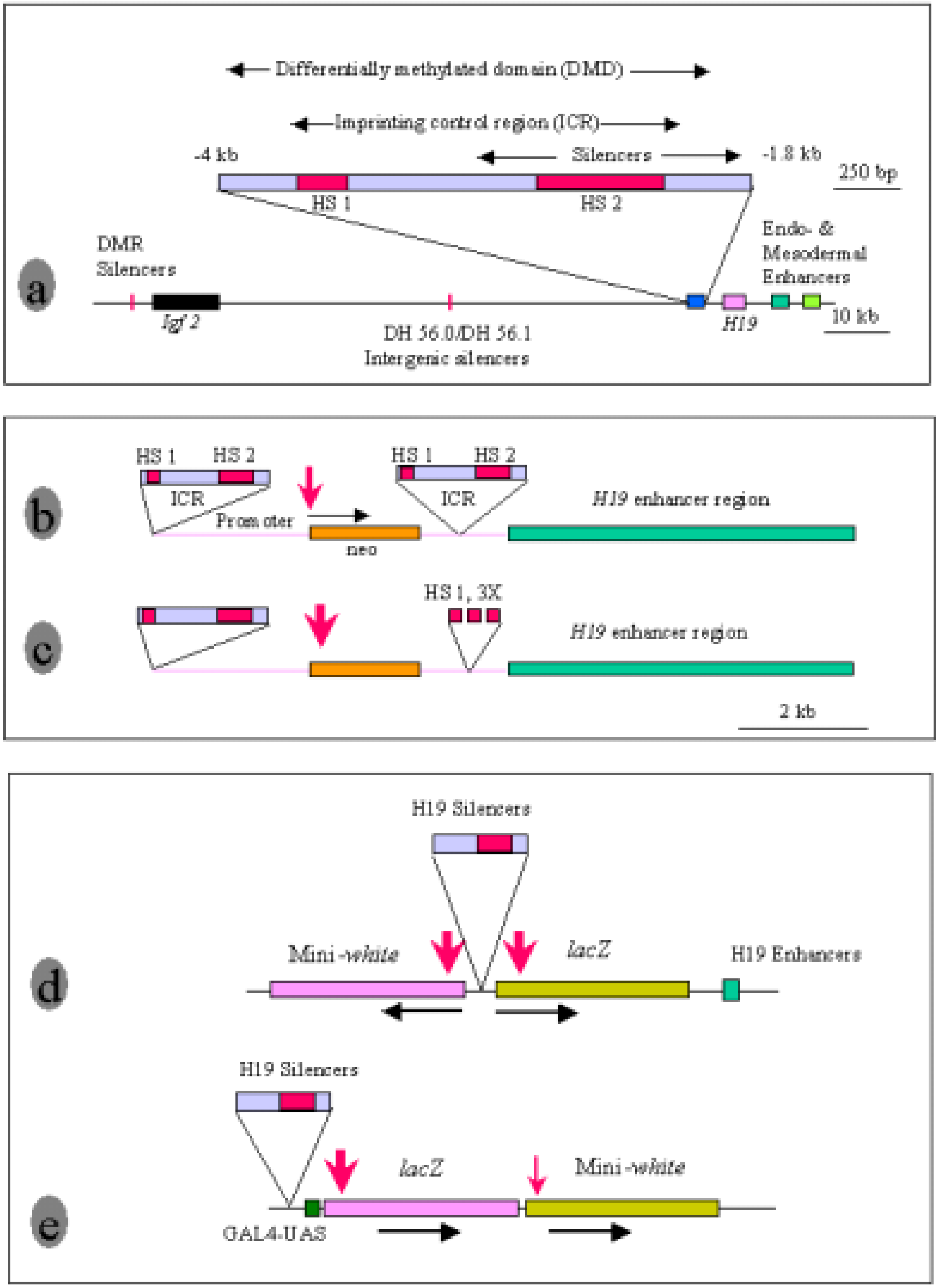

**FIGURE 1.** Insulator and silencer functions of ICR in episomal assays and in transgenic *Drosophila.* (a) Shows the relative position of endodermal and mesodermal enhancers [13,14,39], differentially methylated domain (DMD) [2,3], ICR [5], silencers in the paternal allele [6,8], *Drosophila* [4], and DNase hypersensitive (DH) sites 1 and 2 in the maternal allele [9,11,12,15,42], constitutive evolutionarily conserved DH sites (DH56.0/DH56.1) in the intergenic silencer region [40,41], *Igf2* silencers [39], and the genomic organisation of *Igf2* and *H19* genes in mouse. (b) and (c) Demonstrates the insulator activity of ICR by positioning it between promoter and enhancer; the ICR extends up to –3.8 kb relative to the *H19* transcription start site (b) or multiple copies of one of the DH sites (site 1) inserted in tandem (c) between *neo* promoter and *H19* enhancers [11] (~6 kb region). (d) and (e) The silencing effect of *H19* ICR in *Drosophila* transgenics [4]. A part of the *H19* ICR (silencers in (a)) was inserted between *Drosophila* mini-*white* transformation marker and *lacZ* reporter gene and was driven by *H19*-specific endodermal enhancers inserted at the 3′ end of *lacZ* (d) or a part of the ICR was juxtaposed to Gal4-upstream activating sequences immediately upstream of the mini-*white* and *lacZ* genes in tandem (e). The transgenic lines were created by P-element–mediated genomic integration. The thickness of the arrows (downward and red) indicates the degree of transcriptional repression; horizontal arrows show the direction of transcription.

the episomal constructs resulting in transcriptional repression. A similar position-dependent effect could explain the paradoxical silencer activity of ICR in transgenic *Drosophila*.

The transcriptional activation of both *Igf2* and *H19* genes is dependent upon the direct or indirect participation of a set of endoderm- and mesoderm-specific enhancers located several kilobases downstream of the mouse *H19* gene [13,14] (see Fig. 1a). Therefore, both genes on the same chromosome compete for recruiting the common enhancers. However, there appear to be two chromosome-specific regulatory mechanisms operating in *Igf2/H19* imprinting. In the paternal chromosome, this is achieved by DNA methylation-dependent heterochromatization of ICR most probably inherited from the sperm [15] and thereby allowing the enhancers to interact with the regulatory elements of the *Igf2* gene. In the maternal chromosome, the unmethylated ICR interacting with enhancers activates *H19* transcription as well as negatively regulating the distant *Igf2* gene.

A differentially methylated domain (DMD), ~2 kb upstream of the mouse *H19* gene (–2 kb to –4 kb) is hypermethylated in sperm, and these epigenetic modifications are maintained in the paternal allele of the embryo [2,3] (see Fig. 1a). This observation hinted that the signals for condensing the paternal *H19* allele could possibly lie in this region. The first experimental suggestion that a part of this region (–1.8 kb to –3 kb) could function as a silencer came from transgenic experiments in *Drosophila* [4]. The DNA sequences in this region, therefore, in the absence of DNA methylation are capable of inhibiting transcription in a heterologous system. These data, however, could not explain why hypomethylated ICR in the maternal chromosome, in addition to facilitating *H19* transcription, could simultaneously exert a negative effect on the *Igf2* gene in *cis*. In a recent series of reports [9,10,11,12], this has been explained by demonstrating that unmethylated ICR in the maternal allele is an insulator. For *H19* ICR, two DNase hypersensitive sites (at –3.7 kb, HS1, and at –2.2 to –2.7 kb, HS2), each containing two CTCF binding sites, are capable of blocking the transcription of *H19* or a reporter gene in transgenic animals or in episomal constructs in cultured mammalian cells [9,10,11]. Moreover, CTCF has been shown to bind ICR specifically on the maternal chromosome [16].

We first examine whether *H19* ICR is a silencer. Deletion of the ICR (–2. 1 kb to –3.7 kb) or a part of the ICR plus upstream sequences (–1.8 to –3 kb) at endogenous loci and subsequent germ-line transmission of these mutations activates the paternal *H19* gene in the embryo [5,8]. This effect has also been reproduced by germ-line transmission of the deletion (–1.8 to –3 kb) in transgenic animals [6]. Therefore, the ICR on paternal germ-line transmission functions as an *H19* silencer in the embryo. It is noteworthy, however, that such silencer function could not be mimicked in an episomal conformation in cultured mammalian cells [9], presumably due to the lack of germ cell-specific methylation or the lack of position effect (see below). Furthermore, the enhancer competition which is the foundation of *Igf2/H19* imprinting did not operate in differentiated cell lines [17]. These cells, nevertheless, were capable of supporting the insulator function of ICR in reporter constructs [9,11].

One of the intriguing aspects of the experimental insulator model is that in order to achieve maximum repression of a reporter gene the insulators must be placed in a specific orientation between the two promoters that are driven by enhancers [9] or by positioning the ICR between the promoter of a reporter gene and *H19* enhancers [10,11] (Fig. 1b and 1c). In colony-forming assays, DH sites 1 and 2 (each with two CTCF binding sites) together are better insulators than one site alone; in fact, the more the CTCF sites (tandem insertion, see Fig. 1c), the better the insulation [11]. The single role suggested here for the ICR is that it blocks the enhancer from acting upon the promoter, thereby silencing the reporter gene [10,11]. In the following section, we argue that an alternative explanation for the insulator-mediated inactivation of the reporter gene may exist.

The ICR/insulators, residing between 2 to 4 kb upstream of the *H19* gene, must interact with the distant enhancers (Fig. 1a) directly, by looping, or indirectly, by linking via chromatin facilitators (comprehensively reviewed in Ref. 18 and summarized in Ref. 9) on the maternal chromosome. A possible direct interaction through a 11- to 14-kb chromatin loop would engage the enhancers and thereby structurally block/insulate transcription from *Igf2*. In episomal constructs, this contact by looping is less likely because the insulators and the enhancers are often juxtaposed. In such an artificial alignment of two disparate regulatory modules, the ICR might disable the enhancer or the insulators might begin to behave like silencers. These ideas stem from several independent lines of experiments. First, in the chicken genome, a variety of combinatorial modules involving CTCF binding sites juxtaposed to hormone response elements exhibit remarkable flexibility in silencing or activation of genes depending upon the absence or presence of ligands. For example, chicken lysozyme silencers (S –2.4 kb), composed of CTCF binding sites plus TRE (thyroid hormone response element), synergistically activate or silence the transcription depending upon the presence or the absence of thyroid hormone (T3), respectively [19]. On the other hand, a combination of CTCF module and a negative TRE located at the 3′ untranslated region of a gene synergistically activates or represses transcription in the absence or presence of T3, respectively [20,21]. Second, unlike episomal assays, random genomic integration of β-globin insulator (cHS4) at certain chromosomal loci could silence the reporter gene irrespective of its 5′ or 3′ location with respect to the neighboring enhancer [22]. Third, none of the known insulators (cHS4, scs or BEAD-1) was able to activate transcription when placed in the intervening sequences between viral repressor site and the promoter [23]. Other experiments relate to certain unique characteristics of CTCF itself, including extremely diverse DNA sequence specificity, high nuclear abundance as transcription factor SP1, requirement of large flanking sequences for DNA binding (–50 bp), blocking of initiation/elongation of transcription, and the presence of multivalent repressor binding sites [24,25,26,27,28,29,30,31]. A number of different combinations of zinc-finger motifs may determine specific interaction of CTCF with activators or co-repressors [24,27,29, 30,31]. Indeed, a recent report [32] showed that CTCF-mediated transcriptional silencing involved direct binding of one of the autonomous repressor domains with co-repressor SIN3A which in turn could recruit histone deacetylases. The indirect communication between enhancer and promoter by linking through facilitator proteins will therefore be blocked due to CTCF suppresser functions. This scenario might be analogous to the unidirectional silencing by the *gypsy* insulator in *Drosophila*. The *Drosophila* zinc finger protein similar to mammalian CTCF is the *suppressor of hairy-wing* [*Su* (*Hw*)] which silences transcription by directly binding repeated octamer motifs in the *gypsy* insulator. This silencing phenotype is very similar to that of the bidirectional spreading effect of heterochromatin (variegation). However, the specific interaction of Su (Hw) suppressor with another protein, modifier of mdg 4 [mod-(mdg 4)], results in a unidirectional insulation of reporter promoter [33]. It is interesting to note that none of the target sequences for Su (Hw) has yet been shown to be part of the endogenous insulator element, and the *Drosophila* embryos carrying null mutation of *Su* (*Hw*) develop normally except that the females are sterile [34].

The position-dependent localised folding of chromatin resulting in insulation of transcription in cultured cells [9,10,11] could be mechanistically similar to the ICR-mediated silencing in *Drosophila* transgenics [4]. Instead of being placed between the promoter and enhancer, the ICR was buffered by two promoters (mini-*white* and *lacZ*) away from the enhancer (Fig. 1d). Here, the silencing of the two promoters, we suspect, was independent of the enhancers. The enhancer effect on the nearest promoter (*lacZ*) could have been seen provided the ICR was sufficiently away from the promoter, thereby failing to exert its negative effect on transcription. In fact, the position-dependent silencing could occur when a part of the ICR was juxtaposed to the Gal4 upstream activating sequences (Gal 4-UAS, Fig. 1e) suggesting that the enhancer was dispensable for establishing ICR-mediated silencing effect in *Drosophila*. Moreover, the silencing effect was localised because the *white* promoter, compared to that of *lacZ*, was less subject to repression.

An insulator is an evolutionarily conserved neutral boundary element that separates the neighboring chromatin domains and thereby prevents inappropriate activation/repression of promoters residing in two consecutive loops [35,36,37,38]. Such a role in physiological contexts has been supported by the presumed functions of the specialised chromatin structures (scs and scs′) and cHS4 during induction of *hsp70* gene in *Drosophila* and in the developmental regulation of chicken β-globin gene, respectively. An insulator by itself, therefore, is not an activator or an enhancer or a repressor. On the other hand, a variety of silencers are usually found in the viscinity of a gene (upstream or downstream) and their effects are localised, position-dependent, and directed toward the promoter. The alternative possible silencing effect of ICR on transcription of a reporter gene could be tested. First, one must determine the maximum distance between the enhancer and the ICR that could be tolerated to retain the insulator function in full capacity. An increase in linear distance between the two modules (e.g., 5–10 kb) would not be expected to derepress the reporter gene. Second, although ICR has been placed upstream and downstream of the *H19* promoter or reporter gene [10,11], its position-dependent repressor activity has not been critically determined. Therefore, it is imperative to test whether ICR placed sufficiently close to the promoter (as has been shown in *Drosophila* [4]) could repress *H19* transcription in transient assays. Finally, one could test whether CTCF in the episomes failed to bind SIN 3A or recruit histone deacetylases.

The ICR-mediated position-dependent silencing of reporter genes in transfection and transgenic assays proposed here by no means rules out the putative insulator role of *H19* ICR in *Igf2/H19* imprinting. As mentioned earlier and proposed by others [39], the endodermal enhancers can directly interact with ICR through a small chromatin loop (~15 kb) and thereby structurally block the access of RNA polymerase to *Igf2*. One of the ways to verify this possibility would be to address whether a promoter inserted further upstream of the ICR in the intergenic region could be activated on maternal transmission. Alternatively, a more global interdomain interaction through chromatin loops, also based on differential engagement of the endodermal and mesodermal enhancers, could be envisaged. This model [1] could account for other recently described regulatory elements implicated in *Igf2/H19* imprinting. For instance, deletion of a 1-kb evolutionarily conserved intergenic sequence [40] (DH56.0/DH56.1, see Fig. 1a) between *Igf2* and *H19* in a 130-kb YAC transgene resulted in activation of the maternally silent *Igf2* in the skeletal muscle cells and in the tongue of late embryos. Therefore ICR in combination with all the crucial regulatory elements failed to insulate *Igf2* on germ-line transmission of the mutant transgene [41]. Additionally, *Igf2* has its own imprinting center (differentially methylated region 1, DMR 1) located ~3 kb upstream of the fetal promoter. The unmethylated DMR 1 in the maternal chromosome appears to be inaccessible to the mesodermal enhancers, thereby silencing the *Igf2* expression [39]. In the chromatin looping model, the communication between the intergenic site and enhancers or *Igf2* silencers would critically determine activation of the two genes. Once such interdomain contacts via known regulatory sites are considered, the role of the endodermal enhancers would be to open up the chromatin in the ICR region of the maternal chromosome by preventing the co-repressor binding to CTCF critical for activation of the *H19* gene. This is consistent with the extensive nuclease sensitivity of this region in the maternal chromosome [9,12,15,42].

In summary, the experimental insulator model does not rule out the possibility that ICR functions as a repressor in an episomal context. Indeed, the mechanistic basis (localised folding of chromatin) of this repression could be similar to that of the silencing effect observed in transgenic *Drosophila*. The key to our understanding the dual regulatory role of ICR might rest upon the identification of the *in vivo* and *in vitro* modifiers of CTCF. Recent identification of silencers upstream of the *Igf2* gene and at the intergenic region point toward the possibility of more global interdomain interactions between enhancers, activators/silencers in *Igf2/H19* imprinting.

Finally, it is of interest to examine whether a specific chromatin organizer like an ICR is a general feature of imprinted loci required for the coordinated regulation of the genes therein. Indeed, the dual regulation by a specific chromatin organizer like ICR is not unique for *Igf2/H19* genes. Monoallelic expression of a number of genes at human 15q11-q13 is regulated by several kilobases of heterochromatic imprinting center (IC) in Prader-Willi and Angelman syndromes [43]. Additionally, many of the structural and regulatory features of recently identified reciprocally imprinted *Dlk1/Gtl2* are similar to those of *Igf2/H19* [44,45]. While tissue-specific enhancers, silencers, DMRs, and matrix attachment regions are integral components of imprinted loci, it is difficult to conceive both temporal and coordinate regulation of imprinted genes without considering interaction between neighboring chromatin loops. DNA methylation-dependent localised condensation of a specific chromatin domain (loop) which increases the packing ratio [46] (the ratio of the length of the extended DNA and folded structure) could have a decisive role in regulating the dynamics of such interactions. The loosely packed 30–40 nm solenoid fibres [47] (6 nucleosomes/turn) could be converted to highly compact interdigitated solenoid [48] (14 nucleosomes/turn) in the heterochromatin loop. Biochemically, intrinsic DNA sequence-specific constraints in addition to localised base methylation and covalent modification of histone tails by acetyl-, phosphate-groups, or ubiquitin would determine the stability of such structures. In such a scheme, CTCF binding of unmethylated DNA would be expected to prevent heterochromatization, thereby maintaining a readily accessible chromatin structure.

## ACKNOWLEDGMENTS

We are thankful to Drs. Rainer Renkawitz, Prim Singh, Rolf Ohlsson, and Azim Surani for discussion.

| | | |
|---|---|---|
| Received | February | 22, 2001 |
| Revised | May | 1, 2001 |
| Accepted | May | 4, 2001 |
| Published | May | 18, 2001 |
